# The Contribution of Spanish Science to Patents: Medicine as Case of Study

**DOI:** 10.3390/ijerph17103638

**Published:** 2020-05-21

**Authors:** Mila Cascajares, Alfredo Alcayde, José Antonio Garrido-Cardenas, Francisco Manzano-Agugliaro

**Affiliations:** 1 Department of Engineering, University of Almeria, ceiA3, 04120 Almeria, Spain; milacas@ual.es (M.C.); fmanzano@ual.es (F.M.-A.); 2 Department of Biology and Geology, University of Almeria, 04120 Almeria, Spain; jcardena@ual.es

**Keywords:** Scival, patents, Spain, bibliometrics, Research and Development (R&D), social returns

## Abstract

Investments in research and development (R&D) and innovation are expensive, and one wishes to be assured that there is positive feedback and to receive guidance on how to direct investments in the future. The social or economic benefits of investments in R&D are of particular interest to policymakers. In this regard, public expense in research, especially through universities, is sometimes being questioned. This paper establishes a measure of how research in Spain, and specifically in its universities, is involved. In this study, we have analyzed all the literature cited in the period 1998–2018 produced by Spanish institutions and which has been cited in at least one international patent, obtaining more than 40,000 publications from more than 160,000 different authors. The data have been surprisingly positive, showing that practically all public universities contribute to this subject and that there is a great deal of international collaboration, both in terms of the number of countries with which they collaborate and the prestige of the institutions involved. Regarding the specific scientific fields in which this collaboration is most relevant, biochemistry, genetics and molecular biology, and medicine together account for almost 40% of the total works. The topics most used by these publications were those of diseases or medical problems such as: Neoplams, Carcinoma, Alzheimer Disease, or human immunodeficiency virus (HIV-1). Oncology was according to the All Science Journal Classification (ASJC) the leading and central issue. Therefore, although the result of basic research is difficult to quantify, when it is observed that there is a return in fields such as medicine or global health, it can be said that it is well employed. In terms of journals from a purely bibliometric point of view, it has been observed that some journals do not have a great impact or relative position within their categories, but they do have a great relevance in this area of patent support. Therefore, it would be worthwhile to set up a rank for scientific journals based on the citations of patents, so the percentage of articles cited in patents with Field-Weighted Citation Impact (FWCI) >1, and as an indicator of scientific transfer from universities or research centres, the transference index in patents (TIP) is also proposed.

## 1. Introduction

Basic needs are all those vital necessities that contribute directly or indirectly to a person’s survival, and among the most basic or subsistence necessities could be considered those of health and food. Science must respond to the needs of society and to global challenges [1]. Scientific progress enables us to have a better quality of life [2,3], for example the field of health [4] provides us with new medication to treat diseases and, if not possible [5], at least to mitigate pain [6]. 

Patents protect inventions that consist of products and processes that can be reproduced and replicated for industrial purposes [7]. Companies, laboratories, and individuals can apply for a patent to protect a new technology, sometimes even simply to establish technological boundaries [8]. Whatever the strategic reasons, a patent can be applied for, only if it is for industrial use [9,10]. They are extremely relevant to companies, as they are resources that serve the long-term business. The idea is to keep them in the company for a long period of time. In this way it is possible to develop or invest in a certain line of business, maintaining a certain advantage or protection against the competence. As a major source of new technology generation in developing and transition countries, universities and R&D institutions have played an increasingly active role in the technological innovation, technology transfer and commercialization of intellectual property resulting from research efforts, that finally contribute to the economic, social and cultural development of countries.

Although it is clear that patents are the engine of the industry, in the case of the biotechnology industry that has an impact on the manufacture of drugs such as vaccines [11] or some other medicines, they have a short-term impact on our health or well-being. The transfer of knowledge from research carried out in universities or research centres to the industrial sector is very complex and generally not immediate, but an important indicator is its impact on the number of patents.

Reviewing the research on the properties of the academic literature cited in the patents, it is fair to mention that one of the first works in this regard is the paper of Francisc Narin and Elliot Noma in 1985 [12]. They focused their analysis on 275 biomedical journals and the biotechnology patents in the US Patent Office classification system. As interesting data, they use as reference time for the citations the first eight years after the documents are published. The main conclusion was that science and technology were converging in key high-tech areas. Another very interesting line of research is the study of the patents that are cited by the patents [13,14], highlighting that scientometric assessments, especially of industrial activity, should include patent statistics.

Peter Collins and Suzanne Wyatt [15] studied genetics patents in the U.S. patent system granted during 1980–1985. Although the data are old, they showed that the average citations per patent to papers in basic research journals depending of the applicant country varies between 1 to 10. Another interesting data was that the age distribution of journal citations in patents granted can reach 25 years. Despite the fact that the literature in this field is extensive, focusing on the field of medicine, it is worth mentioning that it has been analyzed in specific fields since 1998 in Gastroenterology research in the United Kingdom [16] till more lately in 2019 in Cardiovascular disease research in Brazil [17]. 

Scientific innovation is determined by science and technology which together determine the way forward, thus, research documents and patent literature can be used to characterize scientific and technological research in a quantitative, automatic and visual way [18]. Recent studies suggest the need to improve collaboration among private and public sectors and health care organizations in research and patent activities [19]. This sort of analysis, from scientific literature and patent search data, has shown as an example that bioinformatics technology is a valuable strategy to modify, synthesize, or recombine existing antimicrobial peptides to obtain drugs against tumors with high activity and low toxicity [20].

There are some works that highlight this issue by analyzing the patents in the field of biotechnology in Spain but in a very short period of time, from 2000 to 2007 [21], or in Brazil in a longer period of time, from 1975 to 2010 [22]. In both studies, they are analyzed from the point of view of patents and not from the point of view of the science that supports them in the form of scientific publications. In any case, this work focuses on the patents obtained by these countries. In the case of the study of Brazil, only 163 patents were international, that is, from the online at World Intellectual Property Organization (WIPO) for the period from 1997 to 2010 [22]. The study of Spain shows a scarce production of patents in biotechnology, compared with European countries with similar scientific and economic capacity, which indicates a deficit in the capacity to absorb the production of new technologies generated in the public scientific sector [21]. In Spain, some recent studies in the field of medicine propose the inclusion of patent databases such as Lens.org for the assessment of the quality criteria of scientific publications [23].

The University must contribute to social and scientific progress and therefore must respond to the demands and needs of the society in which it is embedded [24,25]. Spain accounts for 87 universities of which 50 are public [26]. With respect to public universities, there is a long tradition, as the first public Spanish university that still exists was founded in 1218, the University of Salamanca (although, the first university was that of Palencia in 1209), and the last one was founded in 1998, the Polytechnic University of Cartagena. As for private universities, the first was founded in 1886, the University of Deusto, and the last three were founded in 2019. In this context, the statistics for Spain are satisfactory, with a population of almost 47 million inhabitants, there’s a public university for every million inhabitants. Thus, if the age group is between 18 and 24 years, that’s four million people of university age, and the rate increases to more than 12.5 public universities per million inhabitants. If private universities are also considered, the data amounts to almost 21.75 universities per million people of university age. Regarding the research funding in Spain, this was 15,000 million euros (€15 billion) in 2018.

On the other hand, the analysis of innovation systems often occurs at a national, aggregated scale, frequently based on surveys or bibliometric data derived from scientific publications such as academic papers and patents [27]. University patenting has grown most rapidly, especially in fast-growing technologies, in which university-business co-patenting is most prevalent. This suggests that rising public investment in university research is paying off, and that university research is industry-relevant [28]. In this sense, if the patents applied for by Spanish universities since they have been registered are analyzed, it can be seen that in the period from 2007 to 2018 there have been 6322, of which 327 were in 2018, the lowest amount in the whole historical period [29].

The objective of this research is twofold. On the one hand, to offer a global perspective of the knowledge transfer carried out by Spanish universities, understood as the influence that their scientific publications have on patents, that is, on those publications that are cited by the patents, and within these, the impact that this transfer has on the field of medicine, since it is one of the most important research activities in Spain. Secondly, a proposal was made to provide an index to classify universities according to their transfer, and in particular the publications cited in patents.

## 2. Materials and Methods

The data have been acquired using scientific databases through the different tools that these databases make available to us. Currently, access to these databases is restricted to the organizations that have subscribed to them, which limits the use of these sources. There are free access sources to access scientific publications, but the quality of the data is not the same as in the sources that are mentioned below. Logically, access to science is limited for some researchers, but the reality is that the dominance of these resources has made them indispensable for the world of research, becoming official data sources at the institutional and governmental level.

Scopus is the database developed by Elsevier that indexes the content of more than 24,600 active journal titles and more than 194,000 books from more than 5000 publishers. Its historical content dates back to 1788, and currently contains over 75 million articles, 1.4 billion references cited since 1970, over 9.5 million conference proceedings, 437 million patents from the five largest patent offices worldwide, 16 million author profiles, and around 70,000 membership profiles. Therefore, this database has been used in considerable bibliometric work in every field of knowledge [30], including medicine [31,32].

Based on the data from Scopus, Elsevier has developed its own research performance analysis tool: SciVal, offering access to the scientific output of more than 230 countries and 14,000 institutions from 1996 to the present. It should be noted that this database has also been used for studies related to the field of medicine [33]. Therefore, the main source of data for this study has been Scopus, obtained through SciVal.

In order to complete data on the ranking of scientific journals has been used:

- SCImago Journal Rank (SJR) indicator. Developed by SCImago from the widely known algorithm Google PageRank™, this indicator shows the visibility of the journals contained in the Scopus® database from 1996 [34].
- CiteScore (Scopus). This recent metric, launched in 2016 by Elsevier, is a way of measuring the citation impact of serial titles. It is an alternative to the JCR impact factor (IF) [35].
- JCR (journal citation reports), is a quality indicator of journals that measures the impact of the journals according to the citations received in the Web of Science in the SCIE (Science Citation Index Expanded) and SSCI (Social Science Citation Index) collections. JCR (Journal Citation Reports) provides a quality indicator of journals that measures the impact of the journals according to the citations received in the Web of Science in the SCIE (Science Citation Index Expanded) and SSCI (Social Science Citation Index) collections [36].

To obtain data under analysis, the following search in SciVal has been used as a starting point: “scientific publications in Spain between 1998 and 2018 filtered by ASJC categories”. Journal classification approaches perform an essential function in bibliometric analysis [37]. ASJC (All Science Journal Classification) categories is the classification of subjects used by SciVal to categorise Scopus sources and the publications of each of those sources (e.g., journals). Each Scopus source can be assigned to one or more categories in the selected subject classification. Initially there are the four major subject areas: physical sciences, health sciences, social sciences, and life sciences. The ASJC classification has 27 categories (see Table 1) which are further subdivided into various subcategories. Note that multidisciplinary belongs to the four subject areas.

**Figure 1:**
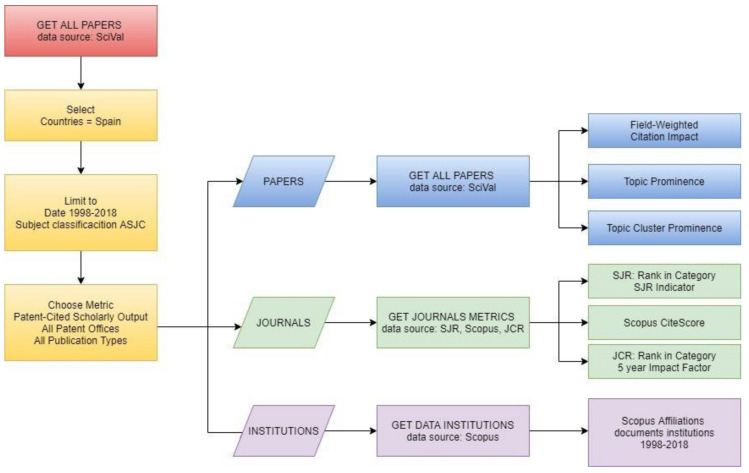
Methodology flowchart. Note: SJR (SCImago Journal Rank); JCR (Journal Citation Reports)

Figure 1 summarizes the methodology. Once the search was performed, it was filtered by the bibliometric marker “Patent-Cited Scholarly Output” for all publication types and for all patent offices. The result provides all publications that have been cited in at least one patent. The coverage of these patents reaches the five largest patent offices: EPO (European patent office), USPTO (U.S. patent office), UK IPO (UK intellectual property office), JPO (Japan patent office), and WIPO (World Intellectual Property Organization).

On the basis of these data, the evolution over time from 1998 to 2018 of the publications that have been cited in patents, the contribution of the authors of these publications to the development of patents, as well as the international collaboration between these authors have been analysed.

In the analysis of affiliations, the source data of the analysis has been completed with data from global publications of each affiliation between 1998 and 2018 based on Scopus. The search has been carried out by affiliation, considering the publications that as an institution have been published in each of the universities or R&D centers under study in this date range.

When analyzing the impact of the journal, it has been chosen to analyze the impact of the journal on JCR, based on data from 2018, obtaining the following metric values:

- SJR category and position within the category (rank SJR). SJR thematic categories corresponds to the classification assigned to each journal indexed in the Scopus database [34].
- Indicator SJR. It expresses the average number of weighted citations received in the selected year by the documents published in the selected journal in the three previous years
- CiteScore. Calculating CiteScore is based on the average citations received per document. CiteScore is the number of citations received by a journal in one year to documents published in the three previous years, divided by the number of documents indexed in Scopus published in those same three years [35].
- JCR category and position within the category. The JCR Category is the thematic category assigned to the journal in the Web of Science and within each category the ranking of the journal is shown, calculated according to the position of the journal in relation to the total of each category. Each journal in JCR is assigned to at least one category and may be classified in more than one category.
- Five-year journal impact factor. This indicator shows the average number of times articles from the journal have been cited in the JCR year over the past five years. It is calculated by dividing the number of citations in the JCR year by the total number of articles published in the previous five years.

When analyzing research topics, SciVal uses so-called Topics. A Topic is a set of documents with a common interest. Topics are based on the grouping of the citation network of 95% of the Scopus content (all documents published since 1996) and are grouped within SciVal based on direct citation analysis using document reference lists, so that a document can belong to only one Topic but as newly published documents are indexed, they are added to the Topics using their reference lists. This makes the Topics dynamic and most of them increase in size over time.

They are obtained from more than one billion citation links between more than 48 million documents indexed by Scopus from 1996 onwards and more than 20 million other non-indexed documents that are cited at least twice. There are approximately 96,000 Topics. Once a year SciVal re-runs the SciVal Topics algorithm to identify newly emerging topics. A combination of the potential for emergence (recent numbers of publications vs. previous years), size of the topic, citations, and funding is considered to rank a new topic. As an example, in 2019, 37 new topics were identified and added to SciVal.

The Topics name is part of the topics cluster name. A topic cluster name is created by adding topics with similar research interests to form a broader, higher-level research area. These topic clusters can be used to gain a broader understanding of the research being carried out by a country, institution (or group) or researcher (or group). Each of the 96,000 topics has been paired with one of the 1500 cluster topics. As with topics, a researcher or institution can contribute to multiple topics, but a topic can only belong to one topic and a publication can only belong to one topic (and therefore to one cluster topic). Clusters topics are formed using the same direct citation algorithm that creates the topics. When the strength of the citation links between the topics reaches a threshold, a cluster topic is formed.

Among all the other possible metrics to evaluate the quality of the journals, it has been chosen the field-weighted citation impact, this is the average number of citations received in relation to the expected ones. Recent studies prove that the FWCI is consistent in different areas of research [38]. Expected citations are calculated for the same year of publication, same type of publication, and same discipline. The benchmark is 1, above which, the expected, and below, it has not reached what was expected. 

## 3. Results

The results achieved from this search, of Spanish scientific papers cited by international patents from 1998 to 2018, have yielded a value of 41,068 cited publications. As expected, almost all these publications are journal articles, more than 96%, being anecdotic the case of the books (Book and Book series) with just over 1% and the conference proceedings with just over 2%. These works have been written by 313,458 co-authors, of which there are 161,046 different authors, identified by their Scopus ID. Most frequently, authors contribute to only one publication, which is the case for 35.5%, those with two are 7.5%, those with three are 3%, those with four are 1.5%, and those with five are less than 1%. By way of exception, there are 50 authors with more than 50 contributions cited by patents. This case study would be particularly interesting to study.

### 3.1. Global Temporal Trend

**Figure 2:**
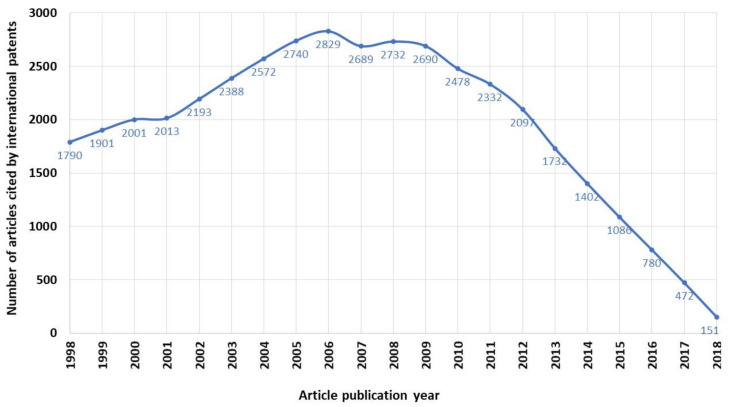
Publications cited by international patents and R&D funding in Spain.

Figure 2 shows the evolution of the articles cited by patents in the period studied. The trend from 1998 to 2008 is very similar, i.e., it seems clear that the greater the research funding, the greater the number of works cited by patents. From this date the trends are different, but it must be clarified that the research does not have an immediate impact on the industry, this trend can be evaluated in the long term, so we can consider that the data up to 10 years ago, should they be representative.

### 3.2. Countries, Affiliations and Collaborations

**Figure 3:**
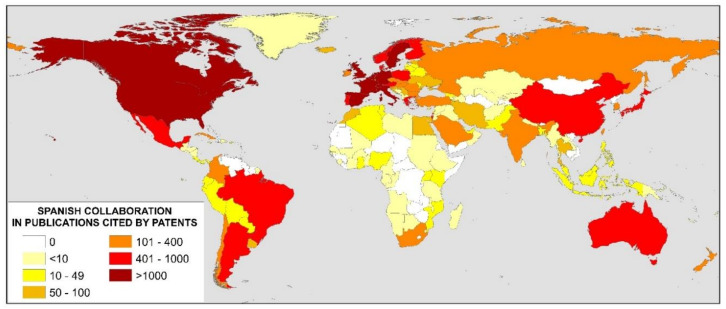
Worldwide collaboration with Spanish publications cited in patents.

The authors of these publications cited in patents belong to just over 5000 institutions around the world, proving a great collaboration with the rest of the world by the Spanish institutions. A total of 165 different countries have been involved, with the USA being the most important with almost 7500 contributions, followed by the UK with around 4200 and Germany with just over 3700. Figure 3 shows a map of Spain’s international collaboration. There is scarce, or almost no, collaboration with African countries, despite the fact that, as will be seen later, the pharmaceutical industry and the area of medicine are very prominent in the domain of patents, and there are very widespread diseases in these areas, such as malaria [39], AIDS, or tuberculosis [40].

**Figure 4:**
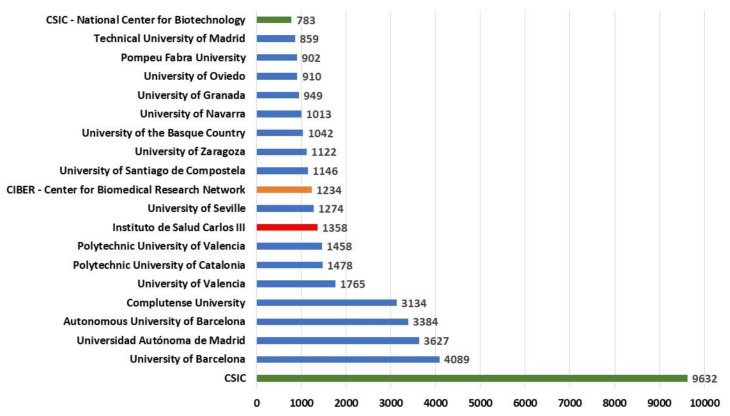
Top 20 Spanish institutions by publications cited in patents. Note: CSIC (Consejo Superior de Investigaciones Científicas).

The top 20 Spanish institutions that have contributed mostly with their publications to international patents are shown in Figure 4. It can be observed as expected that the CSIC, Consejo Superior de Investigaciones Científicas, as the Spanish state agency dedicated to scientific research and technological development, leads this ranking. An example of these studies, which is also widely cited, is a collaboration between University of Valencia, Consejo Superior de Investigaciones Científicas, Beth Israel Deaconess Medical Center, and Harvard Medical School related to the Transcranial magnetic stimulation [41].

Figure 4 highlights the non-university institutions, which include, apart from a specific section of the CSIC, the National Center for Biotechnology, two other autonomous health institutions, the CIBER—Center for Biomedical Research Network and the Instituto de Salud Carlos III. With respect to the universities, two of the first four stand out: The University of Barcelona and the Autonomous University of Barcelona; and two in Madrid, the Autonomous University of Madrid, and the Complutense University. In fifth place is the University of Valencia, but already distant in the number of publications. In this ranking it is remarkable that there are no other research agencies such as CIEMAT (Energy, Environmental and Technological Research Center) that in this period only appear with 251 publications cited, almost as small universities, as the University of Almeria with 210 publications cited in patents. Interestingly, these two institutions cooperate extensively in research, perhaps due to the proximity of one of the CIEMAT centres, Plataforma Solar de Almeria, to the aforementioned university, e.g., they have investigate solar reflector materials degradation due to the sand deposited on backside protective paints [42].

With the aim of having a metric of the impact of the scientific production of the universities in its transference with respect to the patents. The transference index in patents (TIP) is proposed, and this is calculated as a percentage of publications cited in patents over the total number of publications indexed. Table 2 shows this index calculated for the Top 20 Spanish universities. Those that are above 5% are considered outstanding, excellent between 4% and 5%, very good between 3% and 4%, good between 2% and 3%, average < 2%. 

This TIP index shows that among this top 20, 4 universities are in the range of outstanding, apart from the three most productive, now included in this category is the University of Navarra (5.24), which is a private university. In the range of excellent there are two universities: Complutense University (4.74) and Pompeu Fabra University (4.66). Further, in the rank of very good, we find eight universities.

What is surprising at first glance is that the most technological universities are not necessarily the best at this transfer rate: Polytechnic University of Valencia (3.77), Polytechnic University of Catalonia (2.85), and Technical University of Madrid (2.31).

**Figure 5:**
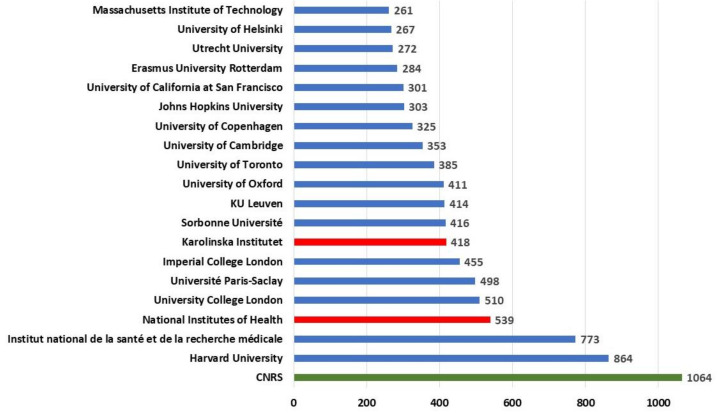
Top 20 Foreign institutions that collaborates with Spanish institutions.

In Figure 5, the top 20 non-Spanish institutions that participate in these works have been represented the ones with which most collaboration takes place. The collaboration of Spanish institutions is especially remarkable with the CNRS (Centre national de la recherche scientifique) in France with more than 1000 works. In addition, there are three other institutions in this country in the top 20: Institut national de la santé et de la recherche médicale (773), Université Paris-Saclay (498) and Sorbonne Université (416). This fact is striking since France was in fourth place in Spanish collaboration. 

Spanish institutions collaboration with foreign countries is significant, as 21,136 of the total number of contributions analysed are of Spanish authorship only, representing 51% of the works. Therefore, in broad terms, this means that half of the contributions cited in patents are in collaboration with foreign institutions. However, when it concerns universities, the percentage is significantly lower Table 2 shows the contributions of each university without collaboration. If the data are analyzed in relative terms, in terms of percentages, the University of Murcia has the highest percentage without collaboration with 41% and the lowest, with 5%, is the Pompeu Fabra University. But the important data is the average, which is 25%. So, only one of each four contributions cited in patents is authored by a Spanish university without collaboration.

Concerning the collaboration with the USA, the first place is taken by Harvard University (864), followed by the National Institutes of Health (539). With the UK, the institutions are universities, University College London (510), Imperial College London (455), and the University of Oxford (411). It is striking that in this top 20 of international collaboration no German institution exists, even though Germany is the third country in terms of international collaboration for Spain. Finally, it should be noted that, apart from the CNRS, the only two non-university institutions carrying out research in the field of health are the aforementioned Institut national de la santé et de la recherche médicale (France) and the Karolinska Institutet (Sweden).

### 3.3. Top Journals Used for the Spanish Publications Cited in Patents

The Spanish scientific studies that have been cited in patents have been published in 4579 different journals. In Table 3, the top 20 of these journals are reported, together with some of their bibliometric indicators. It can be seen that these journals are mainly in the chemical or medical field, with the clear exception of the highly recognized multidisciplinary journals such as Proceedings of the National Academy of Sciences of the United States of America (PNAS), PLoS ONE, or Nature. As an anecdote, there is only one article from the journal Science in this list of journals.

About the metrics of the Top 20 journals obtained, most of them belong to the first quartile (Q1), 17 of the 20 analyzed, but only four are the first of their category. Of the many metrics that can be used to analyze the journals, the field-weighted citation impact has been used, as mentioned, if the publications are above the value of 1, it is more than expected. In our case, the average of all the publications analysed is almost 4 (3.97), and 27,888 of the 41,068 papers analysed are above 1, i.e., 68%. 

Three indices have been chosen to assess the articles published in the top 20 journals: field-weighted citation impact, top 10% topic, and top 10% topic cluster. The Field-Weighted Citation Impact, a Scopus-specific metric value, allows users to measure whether publications have exceeded the percentage of citations expected from them, considering the year of publication, the type of publication and the discipline. The benchmark is 1, so that higher values meet the publication’s expectations and lower values below 1 do not.

**Figure 6:**
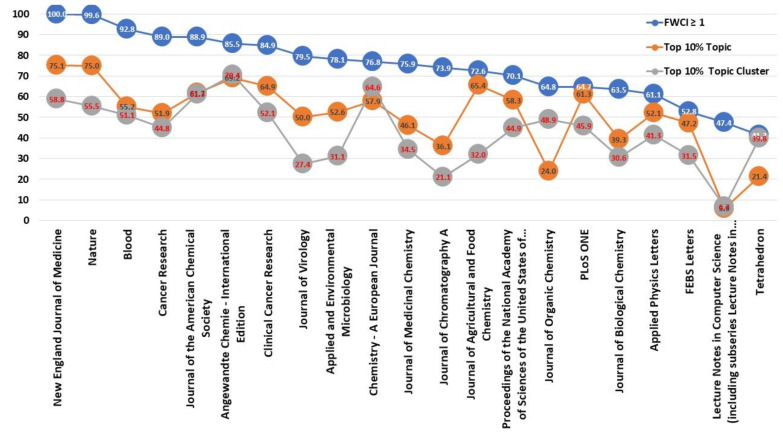
Percentage of articles Field-Weighted Citation Impact (FWCI) ≥ 1, Top 10% Topic y Topic Cluster.

Figure 6 shows the percentage of publications in each of the journals that were above 1. The data show the different percentages achieved, with the New England Journal of Medicine standing out as all the articles published exceeded the value of 1. Between 90% and 100% there are two other journals Nature and Blood, followed by the rest of the journals that are above 50%, only two titles (Lecture Notes in Computer Science, including subseries Lecture Notes in Artificial Intelligence and Lecture Notes in Bioinformatics and Tetrahedron) do not exceed 50%. Based on these data, the majority of publications not only contribute to the development and advancement of science, but also have a direct application in knowledge transfer by being cited in patents. They have surpassed the perspectives expected from them and have had a practical application in research transfer.

The second index considered was the percentage of publications within each journal that contributed to the top 10% of topic and topic cluster. Being in the Top 10% of these values is indicative of the momentum of the Topics that have been assigned to these publications, thus promoting the visibility of these fields of research. Journals such as the New England Journal of Medicine or Nature place more than 75% of their publications in the top 10% of topics, as well as Angewandte Chemie—International Edition, Chemistry—A European Journal or Journal of the American Chemical Society and Angewandte Chemie—International Edition, which place more than 60% of their publications in the top 10% of topic clusters.

### 3.4. Subject Area Classifications of the Publications Cited in Patents

Although the journals are an early indicator of the topics covered, if one uses the classification of the database itself, namely the all science journal classification (ASJC) field name, these contributions appear in four subject areas, which in turn are divided into the 30 categories indicated in Table 1, and this classification allows a third level. This is done by in-house experts when of the serial title is set up for Scopus coverage; the classification is based on the aims and scope of the title, and on the content it publishes. If the distribution of the scientific output by the All Science Journal Classification (ASJC) are analysed regarding the distribution in the four subject areas, the one that contributes most is physical sciences with 44%, followed closely by life sciences with 38%, in third place health sciences with 16%, in fourth place social sciences with less than 1% as expected. The works in the multidisciplinary category have not been attributed to any subject area, being overall 1%. Note that this scientific production refers to the whole, i.e., it includes articles, books, and proceedings.

**Figure 7:**
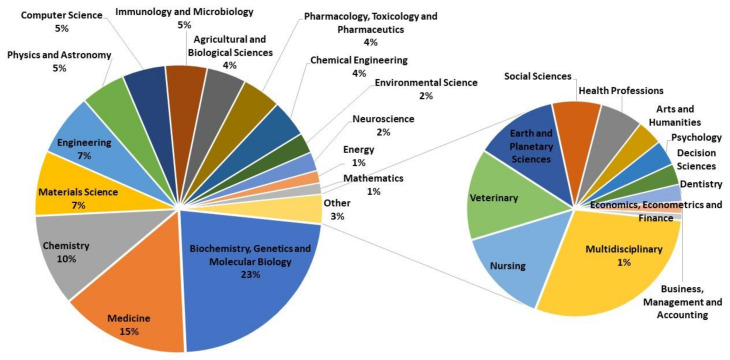
Distribution of the scientific output by ASJC (articles, books, and proceedings).

**Figure 8:**
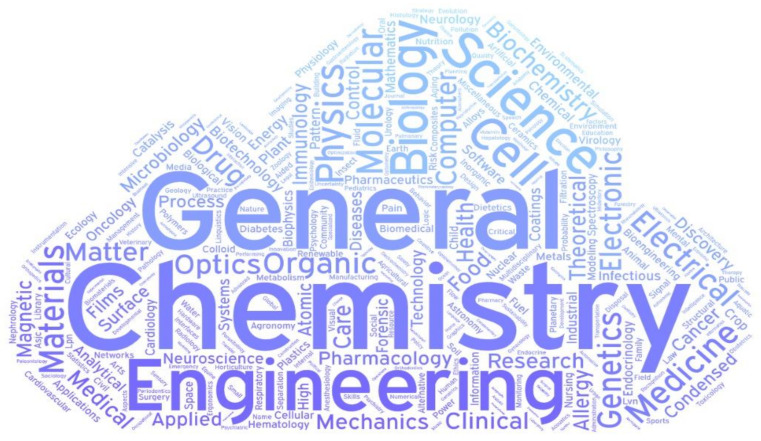
Cloud Word of Topic cluster names.

If the studies are analyzed by subject area classifications, Figure 7 is obtained. The highest percentage of studies is biochemistry, genetics and molecular with 23%, followed by medicine with 15%, and then chemistry with 10%. This means, for example, that of the total number of Spanish scientific output cited in patents, 15% are classified in the field of medicine. The other categories are already below 10%. Figure 8 shows a cloud of words made with the subcategories of the ASJC in order to establish a visual comparison.

### 3.5. Topics of the Publications Cited in Patents

**Figure 9:**
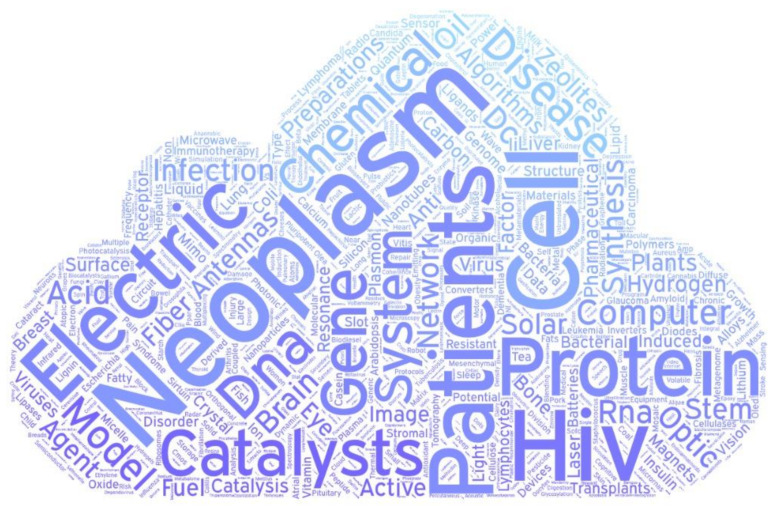
Cloud Word of Topic names.

The topics covered in all these papers could be summarized by two indexing fields: topic cluster name, and topic name. Table 4 lists the first 20 Topic Cluster names and Topic names. In the Topic names, the topics of medicine and biochemistry are more present, but some of other areas such as algorithms, plants, or solar cells are present. On the other hand, in the Topic names no longer appears anything of these other areas, and they do appear diseases apart from neoplasms, appear terms like carcinoma, Alzheimer Disease, or HIV-1. To establish a visual comparison, the topic names have also been represented in a cloud of words in Figure 9.

## 4. The Contribution of Spanish Science to Patents: The Medicine Area 

### 4.1. Temporal Trend in the Medicine Area

Although civil society is sometimes critical of medical patents, as universal access to medicines is understood from a human point of view. However, the WHO (World Health Organization) itself is in favor of this system, since it is clear that, after basic research, they have to be manufactured, and in order for them to be affordable, investment has to be made in their manufacture, which is determined by exclusivity or patents. Furthermore, the WHO itself makes it clear that it is possible to develop many medicines that are patentable (i.e., that meet the requirement of novelty and inventive step), but this does not mean that they add value to existing medicines. According to the data of the IQWiG (Institute for Quality and Efficiency in Health Care—Institut für Qualität und Wirtschaftlichkeit im Gesundheitswesen), of all the medicines that are patented annually, only 10% add great value to what already exists, and only 17% add considerable value, i.e., only 27% of these medicines should be incorporated into the health system.

In the previous sections, a ranking of universities has been established according to their transfer, but, although the Spanish university is not singularly specialized, except as mentioned for certain technical universities. It is necessary to establish a ranking by areas of knowledge. In this way it will be possible to know the transference and the relevance of a university in a specific area. 

**Figure 10:**
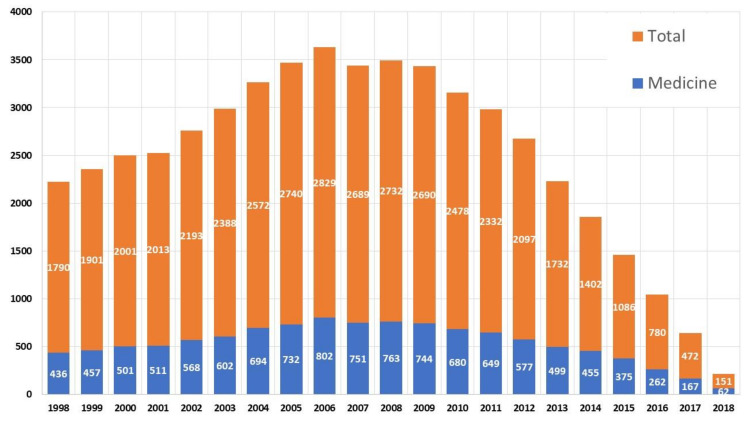
Medicine category display in relation to the total number of publications cited in patents.

In this study, the publications classified within the category of medicine only are 11,287, but there are about 4100 that are also indexed in other categories. If we compare the field of medicine with the total, we can see that it has been very stable over the years. In Figure 10, it can be seen how scientific works classified in the category of medicine have always been at least 20% of the works cited by patents.

### 4.2. Medicine Transference Index in Patents for Spanish Universities

The Spanish scientific output developed by the institutions in the field of medicine cited in patents is reflected in Table 5. Broadly speaking, it can be seen that they are basically the same institutions as the general transfer, with some exceptions of universities, which do not have medical faculties and do not appear in this ranking. If one observes the last column of the table, the explanation is easy: medical research accounts for a very high percentage of the publications cited in patents. The table has been ordered according to this percentage, with the University of Navarra reaching more than 50%. It can be seen that this percentage decreases in accordance with the vocation of each university, so that the last ones in this ranking are technical universities that do not have a medical faculty, and this scientific output is due to collaboration with other institutions.

### 4.3. ASJC Clusters and Relationship Network

**Figure 11:**
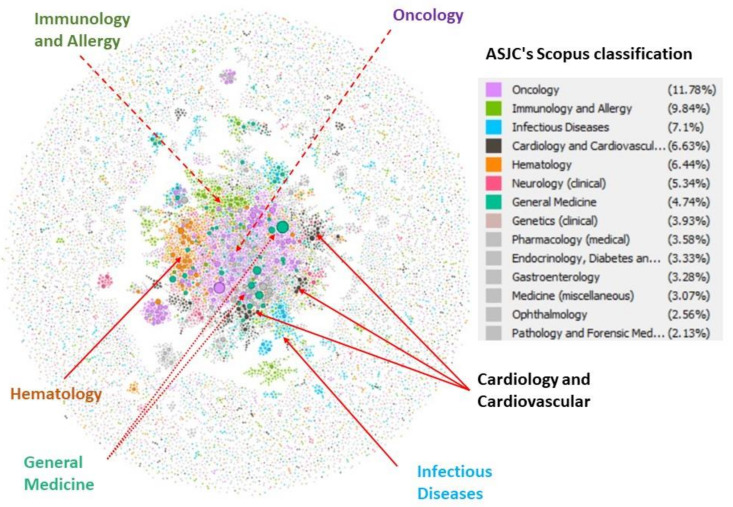
Relationship between publications that are cited in patents in the field of medicine according to the subcategories of medicine of the ASJC.

So, to this point, the above information is that which can be extracted more or less directly from the databases analysed. In this section, the aim is to detect in an independent way, and from the published studies, if the scientific fields of medicine described in previous sections, have any relation between them, that is to say, if they can be grouped in scientific communities or clusters. For this purpose, the bibliometric information of all these works have been downloaded with the Scopus API. If an analysis of data is made with the Gephi software of the network of relationships between the publications that are being analyzed on medicine. Figure 11 shows the relationship found between all the contributions, where each dot is a publication, and the line that joins two dots is the relationship it has for having been cited by that publication, the thickness of the dot indicates the number of times that publication is cited by the others. There is an outer circle of publications, which have no relationship with the others, that is, they would be publication that have been used in the references of some patents in the field of medicine, but which have no relationship with any other publication of this analysis. However, those that are linked to others, are publications that in addition to having been cited by patents, are related to others of this selection of publication. This means that these are more central publication that have been cited by patents, but they have also contributed to opening a line of work in this particular field for research itself since it is related to the other publication. In Figure 11, the publications have been colored according to the ASJR category assigned by Scopus. One can appreciate that they dominate oncology (11,78%), immunology and allergy (9.48%), infectious diseases (7.1%), cardiology and cardiovascular (6.63%), hematology (6.44%), neurology (clinical) (5.34%), and general medicine (4.74%). The oncology category has a central role in this relationship. On the other hand, it is seen that general medicine is widely spread throughout the network, as expected, since it has a direct relationship with all other medical disciplines. This is also the case, although to a lesser extent, with cardiology and cardiovascular.

### 4.4. Cluster Detection Indenpent Analysis

**Figure 12:**
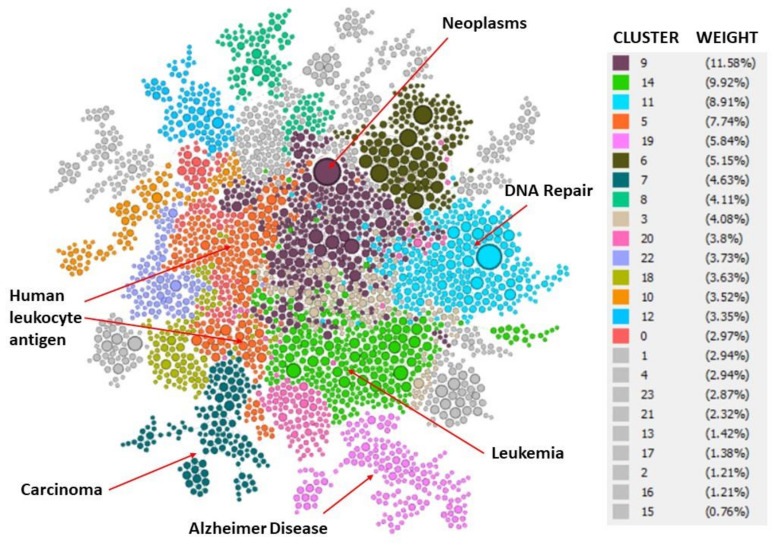
Network of the relationship between publications that are cited in patents in the field of medicine according to the subcategories of medicine of the ASJC.

In a second analysis, the relationships between the publications analysed will be detected. This analysis is independent of the ASJC’s Scopus classification done in previous section. In this case the analysis was done with a cluster detection algorithm that contains the software Gephi. Thus, the clusters have been obtained according to the relationships that exist between the publications. Figure 12 shows a color-coded according to the twenty-two clusters cluster obtained. The weight of the cluster reflects in ratio the significance of this set of publications in the whole network of relations. Once the clusters are established, all the keywords are extracted from all the publications in that cluster. Then, the frequency of each keyword that is found in each cluster is calculated as an index of its importance within that cluster. Table 6, Table 7, Table 8, Table 9, Table 10 and Table 11 show a list of the main keywords for the leading clusters found, up to 5% of weight. The proposed name for each cluster was made according to the keywords of this cluster.

The advantage of this second analysis is that it allows to detect which specific medical topics are being transferred to patents. Thus, the leading topics obtained were: neoplasms, leukemia, DNA repair, human leukocyte antigen, Alzheimer disease, and carcinoma.

### 4.5. Top Journals Used for the Spanish Medicine Publications Cited in Patents 

Finally, these works have been published in specialized medical journals, and it is worth highlighting which have been the most used by patents in the field of medicine. Table 12 shows the most used journals, where the JCR categories and their ranking in 2018 and their five-year impact factor are also shown. The journals are mostly in the category of oncology (six of them) and Hematology (three of them). These journals mostly occupy relevant positions in their category, being 17 of them Q1, 2 of them Q3, and one Q4 (Drugs of the Future). This last journal is noteworthy because it is an atypical case, journals that are little valued by the scientific community, since the impact and position are based on the number of citations received for other scientific work, while here, they appear in a ranking of publications used in patents. Of course, the title of the journal itself has a strong emphasis on technology transfer. A bibliometric reflection on this work would be whether a ranking of journals cited in patents would be worthwhile, that is, as an indicator of scientific transfer fed by the sector itself and in which the university and research centres can also be involved.

**Figure 13:**
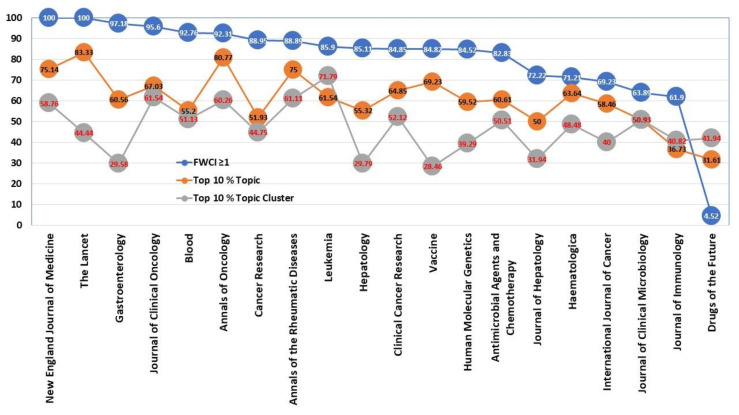
Percentage of articles at Topo 20 medicine journals: FWCI ≥ 1, Top 10% Topic and Topic Cluster.

The analysis made to assess the articles published in the Top 20 journals cited in patents is now made in the case study of the category of medicine for Percentage of articles at Top 20 medicine journals: FWCI ≥ 1, top 10% topic and topic cluster. Figure 13 shows that the New England Journal of Medicine and the Lancet have all their articles above the expected citation value (100 % of FWCI ≥ 1). Four other journals also reach values between 90% and 100%, these are Gastroenterology, Journal of Clinical Oncology, Blood, and Annals of Oncology. Another eight journals are above 80% and all are above 50%, except Drugs of the Future with a very low percentage (4.52%).

According to the percentage of articles that are in the top 10% in topic, it is observed that there is a gradual increase among the journals, and that this ranking is led by journals with more than 75%, which are in order: The Lancet, Annals of Oncology, New England Journal of Medicine, and Annals of the Rheumatic Diseases. Then there are 13 journals with more than 50% and only two below 50%: Journal of Immunology, and Drugs of the Future. The topic cluster has an even lower grading, and there is no journal above 75 %. However, above 50% would be nine journals, where the first four are now: Leukemia, Journal of Clinical Oncology, Annals of the Rheumatic Diseases, and Annals of Oncology. This last journal is the only one that is among the first four in the three rankings analyzed FWCI ≥ 1, top 10% topic and topic cluster.

## 5. Conclusions

Universities and, by extension research centers or agencies, have the duty of producing knowledge, which is generally measured by their scientific production in the form of publications, which, if they are of good quality, are included in international databases that serve as a basis for future research or technological development. Today, this mission is intended to be extended to the solving of society’s problems in general and specifically to the demands of the industrial segment. This new purpose has to date not been easily measured except in the form of patents that universities themselves have developed or applied for. However, this last aspect remains the most important aspect of basic research, which is probably the one that involves the greatest amount of funding. This study has been motivated by the need to understand the role of public research in the development of industry, which is reflected in the contribution to the number of patents. The aim is to address an important gap in the research system by proposing the dilemma of applied research versus basic research carried out in universities and research centres.

This study sets out a methodology to assess the impact of university research on the patent system by analysing the global impact of universities on international patents. In order to evaluate this parallelism, a methodology is established to relate the contribution of Spanish scientific production to international patents based on their citation in these patents. The study was carried out at a global level, but it has been reduced to the field of medicine since the high percentage (20%) of studies cited in patents related to this scientific field.

It has been observed that overall investment in research means an increase in the number of publications that have been cited in patents. Therefore, a direct relationship between funding and transfer is shown. At the same time, international collaboration amongst Spanish authors of these publications is a constant, as shown by the high level of collaboration with countries such as the USA. UK, or Germany at a global level and with France in the field of medicine. Apart from the leading role of the public research body (CSIC), the universities are the institutions that produce applied research and are cited in the patents. A method has been presented that allows the classification of universities based on the relationship between their overall scientific production and the production applied to patents. The results obtained allow to observe that the universities with a TIP (transference index in patents) higher than 5% (outstanding) are not those that have a mainly technological profile, as it would be reasonable to think. However, in the medicine transference index in patents (MED-TIP), it is the universities with medical schools that are positioned at the top of the table.

As an index of where Spanish science is standing out at the transfer level, the Topics and Topic Clusters have been considered. In addition, the highlighted Topics can be used for decision-making in future allocations to research funding. However, the fact that prominence (the topics) represents demand and general visibility should not be lost sight of. It is therefore necessary to support the top 10% topic and top 10% topic cluster indicators. The analysis of the topic and cluster topic has determined networks relating the publications cited in patents both at a general level and from the medical point of view. The clustering of outstanding topics translates directly into the visibility of these publications for the industry sector.

This study shows that public research is fundamental to industrial R&D, as reflected by the number of patents that are based on this knowledge and significantly to R&D in the field of medicine. The leading topics according the ASJC classification were oncology (11.78%), immunology and allergy (9.48%), infectious diseases (7.1%), cardiology and cardiovascular (6.63%), hematology (6.44%), neurology (Clinical) (5.34%), and general medicine (4.74%). In a more detailed and independent analysis, it allowed to determine the leading topics, which were: neoplasms, leukemia, DNA repair, human leukocyte antigen, Alzheimer disease, and carcinoma.

Contrary to the idea that university research generates abstract knowledge that is of poor use to society in general, this study reveals that public research and above all that carried out in universities suggests new products in the form of patents and therefore helps society to advance. Since patents are the basis for industries to develop a product, such research thus reaches society to improve our quality of life.

In short, from the bibliometric point of view, both databases such as Scopus or Web of Science, which provide quality indicators at the publication level, and databases such as JCR or SJR, which quantify the quality of the journals, lack specific indicators that measure the impact of both the publications and their sources in their R&D transfer aspect. Therefore, a ranking of journals cited in patents has been proposed as an indicator of scientific transfer, since it is fed by the industrial sector itself and in which the university and research centres can also be involved. Thus, for universities, the TIP (transference index in patents) has been proposed as a long-term indicator of scientific transfer in patents. In spite of the revealed complexity of the problem about the rates of return to R&D, this work opens new perspectives in the field of transfer of both basic and applied science by proposing a ranking for both journals and research centres, all based on the work cited in patents.

## Figures and Tables

**Table 1:** ASJC (All Science Journal Classification) categories.

Subject Area	Subject Area Classifications
**Physical Sciences**	Chemical Engineering
	Chemistry
	Computer Science
	Earth and Planetary Sciences
	Energy
	Engineering
	Environmental Science
	Material Science
	Mathematics
	Physics and Astronomy
	Multidisciplinary
Health Sciences	Medicine
	Nursing
	Veterinary
	Dentistry
	Health Professions
	Multidisciplinary
Social Sciences	Arts and Humanities
	Business, Management and Accounting
	Decision Sciences
	Economics, Econometrics and Finance
	Psychology
	Social Sciences
	Multidisciplinary
Life Sciences	Agricultural and Biological Sciences
	Biochemistry, Genetics and Molecular Biology
	Immunology and Microbiology
	Neuroscience
	Pharmacology, Toxicology and Pharmaceutics
	Multidisciplinary

**Table 2:** Proposed Transference Index in Patents (TIP).

University	*N* Cited in Patents (NCP)	NCP without Collaboration	*N* (1998–2018)	TIP (TIP = NCP × 100/*N*)
University of Barcelona	4089	781	68,392	5.98
Autonomous University of Madrid	3627	314	63,288	5.73
Autonomous University of Barcelona	3384	551	65,910	5.13
Complutense University of Madrid	3134	587	66,136	4.74
University of Valencia	1765	376	52,037	3.39
Technical University of Catalonia	1478	415	51,882	2.85
Polytechnic University of Valencia	1458	339	38,660	3.77
University of Seville	1274	268	37,233	3.42
University of Santiago De Compostela	1146	412	32,766	3.77
University of Zaragoza	1122	273	32,863	3.49
University of The Basque Country	1042	311	40,071	2.80
University of Navarra	1013	315	19,324	5.39
University of Granada	949	316	43,755	2.32
University of Oviedo	910	365	25,656	3.70
Pompeu Fabra University	902	50	19,366	4.70
Technical University of Madrid	859	197	37,155	2.43
Universidad Rovira i Virgili	751	190	19,691	3.81
Universidad de Salamanca	650	114	19,411	3.35
University of Murcia	595	270	20,699	2.87
University of Málaga	539	178	19,589	2.75

**Table 3:** Top 20 Journals and their metrics (Data 2018).

Journal	*N*	SJR Category	Rank SJR	SJR Indicator	CiteScore Scopus	JCR Category	Rank JCR	Impact Factor (5 years) JCR
Journal of Biological Chemistry	507	Biochemistry	37/446-Q1	2.403	3.92	Biology & Biochemistry	1/434-Q1	4.279
		Cell Biology	45/288-Q1					
		Molecular Biology	69/409-Q1					
Journal of Agricultural and Food Chemistry	350	Agricultural and Biological Sciences (miscellaneous)	32/272-Q1	1.111	3.8	Agriculture, Multidisciplinary	3/57-Q1	3.911
						Chemistry, Applied	14/71-Q1	
		Chemistry (miscellaneous)	56/437-Q1			Food Science &Technology	28/135-Q1	
Proceedings of the National Academy of Sciences of the United States of America (PNAS)	321	Multidisciplinary	4/120-Q1	5.601	8.58	Multidisciplinary Sciences	7/69-Q1	10.600
PLoS ONE	292	Agricultural and Biological Sciences (miscellaneous)	32/272-Q1	1.100	2.97	Multidisciplinary Sciences	24/69-Q2	3.337
		Biochemistry, Genetics and Molecular Biology (miscellaneous)	53/242-Q1					
		Medicine (miscellaneous)	474/2836-Q1					
Journal of the American Chemical Society	253	Biochemistry	5/446-Q1	7.468	14.75	Chemistry Multidisciplinary	12/172-Q1	14.491
		Catalysis	1/58-Q1					
		Colloid and Surface Chemistry	1/16-Q1					
		Chemistry (miscellaneous)	7/437-Q1					
Lecture Notes in Computer Science (including subseries Lecture Notes in Artificial Intelligence and Lecture Notes in Bioinformatics)	253	Computer Science (miscellaneous)	131/449-Q2	0.283	1.06	Computer Science, Theory & Methods (2005 is last year available)	62/71-Q4	n/a
		Theoretical Computer Science	96/160-Q3					
Nature	236	Multidisciplinary	1/120-Q1	16.345	15.21	Multidisciplinary Sciences	1/69-Q1	45.819
Journal of Organic Chemistry	233	Organic Chemistry	15/177-Q1	1.607	4.57	Chemistry, Organic	7/57-Q1	4.224
Journal of Medicinal Chemistry	232	Molecular Medicine	18/173-Q1	2.287	1.05	Chemistry, Medicinal	3/61-Q1	6.060
		Drug Discovery	7/167-Q1					
Blood	221	Biochemistry	9/446-Q1	6.065	7.27	Hematology	1/73-Q1	13.206
		Cell Biology	19/288-Q1					
		Immunology	10/216-Q1					
		Hematology	2/133-Q1					
Applied and Environmental Microbiology	196	Food Science	11/301-Q1	1.663	4.18	Biotechnology & Applied Microbiology	33/162-Q1	4.701
		Biotechnology	30/328-Q1					
		Ecology	27/357-Q1			Microbiology	38/133-Q2	
		Applied Microbiology and Biotechnology	11/111-Q1					
Tetrahedron	196	Biochemistry	228/446-Q3	0.709	2.39	Chemistry, Organic	26/57-Q2	2.193
		Organic Chemistry	60/177-Q2					
		Drug Discovery	56/167-Q2					
Journal of Virology	190	Insect Science	2/146-Q1	2.590	4.02	Virology	8/36-Q1	4.259
		Immunology	25/216-Q1					
		Microbiology	15/149-Q1					
		Virology	8/70-Q1					
Cancer Research	181	Cancer Research	11/216-Q1	4.047	6.94	Oncology	21/230-Q1	9.062
		Oncology	14/368-Q1					
Journal of Chromatography A	180	Biochemistry	122/446-Q2	1.188	3.78	Biochemical Research Methods	13/79-Q1	3.741
		Analytical Chemistry	14/117-Q1					
		Organic Chemistry	27/177-Q1			Chemistry, Analytical	15/84-Q1	
		Medicine (miscellaneous)	417/2836-Q1					
New England Journal of Medicine	177	Medicine (miscellaneous)	3/2836-Q1	19.524	16.1	Medicine, General & Internal	1/160-Q1	70.331
Applied Physics Letters	167	Physics and Astronomy (miscellaneous)	29/267-Q1	1.331	3.58	Physics, Applied	31/148-Q1	3.352
Clinical Cancer Research	165	Cancer Research	7/216-Q1	4.965	8.32	Oncology	16/230-Q1	9.174
		Oncology	10/368-Q1					
Chemistry—A European Journal	164	Catalysis	8/58-Q1	1.842	4.77	Chemistry, Multidisciplinary	37/172-Q1	4.843
		Chemistry (miscellaneous)	31/437-Q1					
		Organic Chemistry	8/177-Q1					
Angewandte Chemie—International Edition	159	Catalysis	2/58-Q1	5.478	11.68	Chemistry, Multidisciplinary	17/172- Q1	12.359
		Chemistry (miscellaneous)	12/437-Q1					
FEBS Letters	159	Biochemistry	63/446-Q1	1.849	3.01	Biochemistry & Molecular Biology	160/299-Q3	3.386
		Biophysics	10/133-Q1			Biophysics	30/73-Q2	
		Cell Biology	76/288-Q2			Cell Biology	129/193-Q3	
		Genetics	60/338-Q1					
		Molecular Biology	101/409-Q1					
		Structural Biology	14/53-Q2					

**Table 4:** Top 20 Topic Cluster names and Topic names.

Topic Cluster Name	*N*	Topic Name	*N*
Neoplasms	3062	Neoplasms	1180
Patients	2743	Receptors	828
Catalysts	1614	Proteins	464
Synthesis (Chemical)	956	Cells	463
Models	883	Patients	374
Algorithms	842	Synthesis (chemical)	348
Genes	821	DNA	339
Hydrogenation	795	Carcinoma	336
Zeolites	795	Genes	312
Pharmaceutical Preparations	686	Mutation	304
Proteins	656	Pharmaceutical Preparations	294
Cells	641	Receptor	288
Catalysis	617	Peptides	284
T-Lymphocytes	596	T-Lymphocytes	276
Plants	586	Ligands	269
Bacteria	555	Breast Neoplasms	263
Solar Cells	551	Nanoparticles	258
Immunotherapy	535	Alzheimer Disease	236
Ligands	495	RNA	232
Breast Neoplasms	489	HIV-1	228

**Table 5:** Medicine Transference Index in Patents (MED-TIP).

University	N-MED ^1^	NCP ^2^	*N* ^3^	TIP ^4^	TIP-MED ^5^	% MED-TOT ^6^
University of Navarra	566	1013	19,324	5.24	2.93	55.87
Autonomous University of Barcelona	1690	3384	65,910	5.13	2.56	49.94
University of Barcelona	2001	4089	68,392	5.98	2.93	48.94
Pompeu Fabra University	372	902	19,366	4.66	1.92	41.24
Complutense University	1223	3134	66,136	4.74	1.85	39.02
Polytechnic University of Valencia	568	1458	38,660	3.77	1.47	38.96
Universidad de Salamanca	223	650	19,411	3.35	1.15	34.31
Universidad Autónoma de Madrid	1207	3627	63,288	5.73	1.91	33.28
University of Valencia	568	1765	52,037	3.39	1.09	32.18
University of Santiago de Compostela	312	1146	32,766	3.50	0.95	27.23
University of Granada	235	949	43,755	2.17	0.54	24.76
University of Zaragoza	268	1122	32,863	3.41	0.82	23.89
University of Murcia	133	595	20,699	2.87	0.64	22.35
University of Valladolid	89	426	17,007	2.50	0.52	20.89
University of Oviedo	189	910	25,656	3.55	0.74	20.77
Universidad Rovira i Virgili	124	751	19,691	3.81	0.63	16.51
University of the Basque Country	159	1042	40,071	2.60	0.40	15.26
University of Seville	143	1274	37,233	3.42	0.38	11.22
Technical University of Madrid	52	859	37,155	2.31	0.14	6.05
Polytechnic University of Catalonia	69	1478	51,882	2.85	0.13	4.67

^1^ N-MED Total number of publications classifies as Medicine category (ASJC) cited in patents; ^2^ NCP Total number of publications cited in patents; ^3^ *N* Total number of publications published by the institution in period 1998–2018; ^4^ TIP = NCP × 100/N; ^5^ TIP-MED = N-MED × 100/N; ^6^ % MED-TOT = N-MED × 100/NCP.

**Table 6:** Neoplasms. Cluster (9), weigh 11.58%.

Topic Names	*N* = 335
Breast Neoplasms, Receptor, Epidermal Growth Factor, Adjuvant trastuzumab	27
Receptor, Epidermal Growth Factor, Neoplasms, Antibodies, Monoclonal	21
Multiple Myeloma, Patients, Diagnosed multiple	13
Activated-Leukocyte Cell Adhesion Molecule, T-Lymphocytes, Activated leukocyte	12
Colorectal Neoplasms, Drug Therapy, Colorectal cancer	11
Colorectal Neoplasms, Mutation, Anti-epidermal growth	10
Breast Neoplasms, Receptor, Epidermal Growth Factor, Immunohistochemistry (IHC)	9
Sirolimus, Neoplasms, Mammalian target	9
Breast Neoplasms, Neoplasms, HER3 expression	8

**Table 7:** Leukemia. Cluster (14), weigh 9.92%.

Topic Names	*N* = 287
Leukemia, Lymphocytic, Chronic, B-Cell, Patients, Lymphocytic leukemia	19
Lymphoma, Large B-Cell, Diffuse, Lymphoma, Rituximab cyclophosphamide	18
Lymphoma, Mantle-Cell, Patients, MCL patients	16
Tetraspanins, Cells, Cell migration	10
T-Lymphocytes, B-Lymphocytes, XIAP (X-linked inhibitor of apoptosis protein) deficiency	8
Multiple Myeloma, Plasma Cells, Cytogenetic abnormalities	7
Leukemia, Precursor Cell Lymphoblastic Leukemia-Lymphoma, Phenotype acute	7
Lymphoma, Follicular, Lymphoma, Mantle cell	7
Precursor Cell Lymphoblastic Leukemia-Lymphoma, Neoplasm, Residual, Disease MRD (Minimal residual disease)	7
Liver Transplantation, Liver, Liver allograft	6

**Table 8:** DNA Repair. Cluster (11), weigh 8.91%.

Topic Names	*N* = 258
DNA, Neoplasms, Liquid biopsies	19
DNA Repair, Carcinoma, Non-Small-Cell Lung, Repair cross-complementation	18
Carcinoma, Non-Small-Cell Lung, Receptor, Epidermal Growth Factor, Lung cancers	16
Epithelial-Mesenchymal Transition, Neoplasms, Epithelial-to-mesenchymal transition	10
Breast Neoplasms, Methylation, Suppressor genes	8
Breast Neoplasms, Neoplasms, Cancer subtypes	7
DNA Methylation, Methylation, Whole-genome bisulfite	7
Methyltransferases, DNA, Temozolomide (TMZ)	7
Precursor Cell Lymphoblastic Leukemia-Lymphoma, DNA Methylation, Methylation	7
Urinary Bladder Neoplasms, Carcinoma, Bladder cancers	6

**Table 9:** Human leukocyte antigen (HLA). Cluster (5), weigh 7.74%.

Topic Names	*N* = 224
Neoplasms, HLA Antigens, HLA class	19
Fetal Blood, Transplantation, Blood UCB (Umbilical Cord Blood)	10
T-Lymphocytes, Neoplasms, Cancer immunotherapy	9
HLA-G Antigens, HLA Antigens, SHLA-G levels	8
Lectins, C-Type, T-Lymphocytes, T cells	8
Killer Cells, Natural, Receptors, Natural Killer Cell, Ly49 receptors	7
Receptors, Antigen, T-Cell, T-Lymphocytes, Antigen receptor	7
Dendritic Cells, T-Lymphocytes, Plasmacytoid DCs	5
Interleukin-12, Neoplasms, Gene therapy	5
Receptors, KIR (Killer Immunoglobulin-like Receptor), Killer Cells, Natural, Killer immunoglobulin-like	5

**Table 10:** Alzheimer Disease. Cluster (19), weigh 5.84%.

Topic Names	*N* = 169
Restless Legs Syndrome, Sleep, Patients	15
Tauopathies, Alzheimer Disease, Tau oligomers	12
Platelet-Rich Plasma, Blood Platelets, Intercellular Signaling Peptides and Proteins	10
Deep Brain Stimulation, Parkinson Disease, Microelectrode recording	8
Alpha-Synuclein, Parkinson Disease, Protein α-synuclein	7
Lipids, Lipolysis, Adipose triglyceride	6
Adrenoleukodystrophy, Fatty Acids, Acids VLCFA (Very Long Chain Fatty Acids)	5
Lewy Body Disease, Dementia, Probable DLB (Dementia with Lewy bodies)	5
Phenylketonurias, Phenylalanine, Phenylalanine levels	5
Alzheimer Disease, Amyloid, Amyloid plaques	4

**Table 11:** Carcinoma. Cluster (6), weigh 5.15%.

Topic Names	*N* = 149
Carcinoma, Hepatocellular, Survival, Sorafenib treatment	20
Hepatitis C, Chronic, Ribavirin, Hepacivirus	13
Carcinoma, Hepatocellular, Neoplasms, HCC (HepatoCellular Carcinoma) patients	10
HIV, Hepacivirus, HIV (Human Immunodeficiency Virus) /HCV (Hepatitis C Virus) co-infected	10
Hepatitis C, Liver Transplantation, Recurrent hepatitis	8
Elasticity Imaging Techniques, Fibrosis, Spleen stiffness	7
Hypertension, Portal, Fibrosis, Cirrhotic rats	6
Hemorrhage, Esophageal and Gastric Varices, Acute variceal	4
Hepacivirus, Ribavirin, Direct-acting antiviral	4
Carcinoma, Hepatocellular, Liver Transplantation, Microvascular invasion	3

**Table 12:** Top 20 journal in medicine category. Data 2018

Journal	N	SJR Category	Rank SJR	SJR Indicator	CiteScore Scopus	JCR Category	Rank JCR	Impact Factor (5 years) JCR
Blood	221	Biochemistry	9/446-Q1	6.065	7.27	Hematology	1/73-Q1	13.206
		Cell Biology	19/288-Q1					
		Immunology	10/216-Q1					
		Hematology	2/133-Q1					
Cancer Research	181	Cancer Research	11/216-Q1	4.047	6.94	Oncology	21/230-Q1	9.062
		Oncology	14/368-Q1					
New England Journal of Medicine	177	Medicine (miscellaneous)	3/2836-Q1	19.524	16.1	Medicine, General & Internal	1/160-Q1	70.331
Clinical Cancer Research	165	Cancer Research	7/216-Q1	4.965	8.32	Oncology	16/230-Q1	9.174
		Oncology	10/368-Q1					
Drugs of the Future	155	Pharmacology (medical)	221/261-Q4	0.123	0.08	Pharmacology & Pharmacy	267/267-Q4	0.109
		Pharmacology	283/330-Q4					
Journal of Immunology	147	Immunology	28/216-Q1	2.521	4.41	Immunology	43/158-Q2	5.066
		Immunology and Allergy	21/203-Q1					
Vaccine	130	Molecular Medicine	28/173-Q1	1.759	3.18	Immunolog Medicine, Research & Experimental	78/158-Q2 57/136-Q2	3.293
		Immunology and Microbiology (miscellaneous)	13/49-Q2					
		Infectious Diseases	41/286-Q1					
		Public Health, Environmental and Occupational Health	38/530-Q1					
		Veterinary (miscellaneous)	2/182-Q1					
Journal of Clinical Microbiology	108	Microbiology (medical)	11/123-Q1	2.314	3.65	Microbiology	24/133-Q1	4.183
The Lancet	108	Medicine (miscellaneous)	5/2836-Q1	15.871	10.28	Medicine, General & Internal	2/160-Q1	54.664
Antimicrobial Agents and Chemotherapy	99	Infectious Diseases	29/286-Q1	2.096	4.34	Microbiology Pharmacology & Pharmacy	28/133-Q1 27/267-Q1	4.719
		Pharmacology (medical)	11/261-Q1					
		Pharmacology	21/330-Q1					
Hepatology	94	Hepatology	3/67-Q1	5.096	6.87	Gastroenterology & Hepatology	5/84-Q1	12.795
		Medicine (miscellaneous)	39/2836-Q1					
Journal of Clinical Oncology	91	Cancer Research	2/216-Q1	11.754	11.08	Oncology	5/230-Q1	22.565
		Medicine (miscellaneous)	9/2836-Q1					
		Oncology	4/368-Q1					
Human Molecular Genetics	84	Genetics	27/338-Q1	3.097	4.88	Biochemistry & Molecular Biology Genetics & Heredity	62/299-Q1 32/174-Q1	5.281
		Molecular Biology	47/409-Q1					
		Genetics (clinical)	8/100-Q1					
		Medicine (miscellaneous)	75/2836-Q1					
Annals of Oncology	78	Hematology	3/133-Q1	6.047	8.44	Oncology	9/230-Q1	11.791
		Medicine (miscellaneous)	35/2836-Q1					
		Oncology	8/368-Q1					
Leukemia	78	Cancer Research Anesthesiology and Pain	8/216-Q1	4.518	6.08	Oncology Hematology	14/230-Q1 4/73-Q1	9.679
		Medicine	1/122-Q1					
		Hematology	5/133-Q1					
		Oncology	11/368-Q1					
Annals of the Rheumatic Diseases	72	Biochemistry, Genetics and Molecular Biology (miscellaneous)	6/242-Q1	7.081	9.18	Rheumatology	2/31-Q1	12.692
		Immunology	8/216-Q1					
		Immunology and Allergy	6/203-Q1					
		Rheumatology	1/60-Q1					
Journal of Hepatology	72	Hepatology	2/67-Q1	6.274	9.32	Gastroenterology & Hepatology	3/84-Q1	14.265
Gastroenterology	71	Gastroenterology	1/145-Q1	7.384	7.07	Gastroenterology & Hepatology	2/84-Q1	19.066
		Hepatology	1/67-Q1					
Haematologica	66	Hematology	6/133-Q1	3.077	4.07	Hematology	7/73-Q1	6.931
International Journal of Cancer	65	Cancer Research	18/216-Q1	3.276	6.93	Oncology	51/230-Q1	6.210
		Oncology	24/368-Q1					
